# Massive Hemorrhage Following Acupuncture Treatment in a Neurofibromatosis Type 1 Patient

**DOI:** 10.7759/cureus.47825

**Published:** 2023-10-27

**Authors:** Wei Wei, Letian Yang, Yuyi Zhu, Caihong Liu, Yuliang Zhao

**Affiliations:** 1 Division of Nephrology, Kidney Research Institute, West China Hospital, Sichuan University, Chengdu, CHN; 2 Department of Neurology, West China Hospital, Sichuan University, Chengdu, CHN

**Keywords:** acupuncture, hematoma, vascular involvement, neurofibromatosis type 1, case report

## Abstract

Neurofibromatosis type 1 (NF1) is a genetic disorder involving multiple organs. Vascular involvement is a rare complication among NF1 patients. We report a case of a 59-year-old female NF1 patient who presented with a massive hematoma over the scapular area after undergoing acupuncture treatment. Contrast-enhanced CT and MRI demonstrated a slightly hyperdense mass measuring 24.2 × 10.3 cm in size, and multiple enlarged and tortuous malformed vessels were seen arising from the left subclavian artery. Arterial embolization and subsequent surgical mass resection were successfully performed. This case indicates that minor injuries such as acupuncture-related ones could cause severe hemorrhage in patients with vascular malformation related to NF1. Endovascular angiography and embolization proved to be effective in localizing the culprit vessel and stopping active bleeding in our patient.

## Introduction

Neurofibromatosis type 1 (NF1), also known as von Recklinghausen disease, is an autosomal dominant genetic disorder caused by the mutation of gene NF1 located on chromosome 17, which results in the dysfunction of tumor suppressor neurofibromin [1]. The average global incidence of NF1 is 1/3000 [1]. NF1 is associated with varying clinical manifestations involving numerous organs. The typical manifestation constitutes neurofibroma, which is a tumor forming on the nerve tissue. Other manifestations include cafe-au-lait macules (CALMs), freckles in the armpits or groin area, Lisch nodules, and bone deformities. NF1 is rarely associated with vascular involvement, the incidence of which is not established but is estimated to be 0.4%-6.4% [2]. We present a case of a 59-year-old female NF1 patient with massive back hematoma due to a rupture of malformed tumor vessels after undergoing acupuncture. The patient underwent interventional embolization of the feeding artery and subsequent surgical mass resection. We also engage in a review of the relevant literature and discuss the clinical manifestation and management of malformed blood vessel bleeding among NF1 patients.

## Case presentation

**Figure 1:**
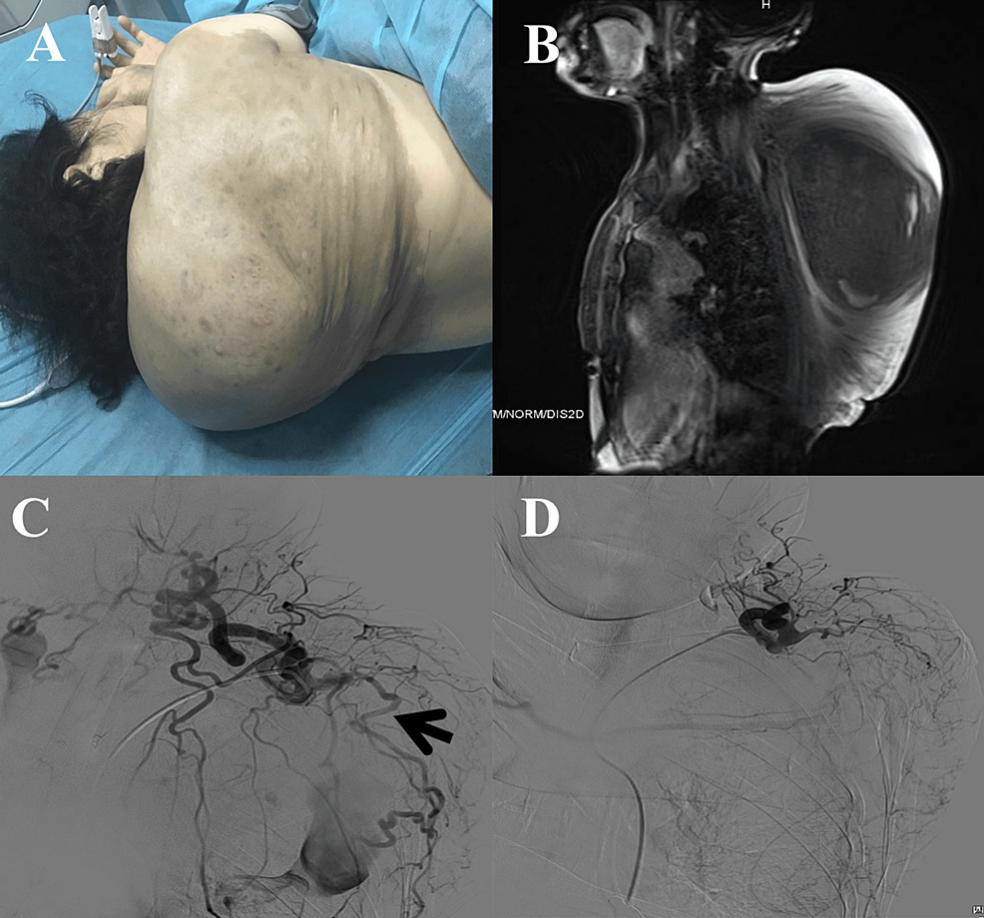
Malformed tumor vessel bleeding treated by interventional embolization in a NF1 patient A: a female NF1 patient presented with a massive back hematoma after acupuncture; B: MRI showing a massive back hematoma (mid-sagittal plane); C: subclavian artery angiography revealed multiple enlarged and tortuous arteries (arrow); D: the culprit artery was embolized MRI: magnetic resonance imaging; NF1: neurofibromatosis type 1

A 59-year-old female with a previous diagnosis of NF1 was admitted to the emergency department with a six-day history of a mass gradually increasing in size on the scapular area after receiving acupuncture treatment for “insomnia” at a clinic (Figure 1A). The patient denied any signs of minor hematoma before the acupuncture treatment. The patient suffered from throbbing pain over the mass. There was no history of fever, dyspnea, and unconsciousness.

At the time of triage, her vital signs were normal. On physical examination, an approximately 30 × 28 × 18 cm mass was palpated, which was soft, brown in color, non-tender, difficultly compressible, and had a well-demarcated border with increased local temperature. More than 10 CALMs were found on the trunk along with multiple subcutaneous nodules (left buttock and bilateral scapular region). Laboratory results revealed that her hemoglobin level was 75 g/L, while fibrin degradation product and D-dimer levels were 62.5 mg/L and 15.25 mg/L respectively. Contrast-enhanced CT and enhanced MRI (Figure 1B) demonstrated a slightly hyperdense mass measuring 24.2 × 10.3 cm in size located in subcutaneous soft tissue (C7-T11 level), and multiple enlarged and tortuous malformed vessels were seen arising from the left subclavian artery.

Compacted red blood cells and recombinant human thrombopoietin were given. The patient underwent left subclavian arteriography via a femoral artery approach (Figure 1C). Digital subtraction angiography (DSA) revealed malformed vessels arising from the left subclavian artery supplying blood to the pseudoaneurysm. Under DSA guidance, the malformed vessels were embolized with polyvinyl alcohol (PVA) particles. Postoperative DSA revealed that the blood supply to the pseudoaneurysm was blocked (Figure 1D). After a few days of supportive treatments, the patient successfully underwent surgical resection of the back mass. Pathology confirmed the diagnosis of plexiform neurofibroma. She was subsequently discharged and followed up later by neurologists.

## Discussion

We discussed a case of a 59-year-old female patient diagnosed with NF1 accompanied by a rare complication of vascular malformation involving the subclavian artery. The massive back hematoma was believed to be caused by the rupture of malformed tumor vessels after receiving acupuncture treatment. Interventional embolization temporarily stabilized the condition and bridged the patient to successful surgical resection of the bleeding neurofibroma.

Among patients with NF1, vascular involvement is uncommon, including renal artery stenosis resulting in secondary hypertension, vessel occlusion resulting in organ infarcts, aneurysms, and arteriovenous fistula resulting in spontaneous hemorrhage. Malformed vessels are usually asymptomatic, but vessel rupture and pseudoaneurysm formation could be fatal. Vascular malformation and spontaneous rupture have been reported in the abdominal aorta, maxillary artery, brachial artery, renal artery, tibial artery, as well as branches of the subclavian artery among NF1 patients [3-9]. However, the prevalence rate of vascular abnormalities is uncertain. It was reported that the incidence of this vasculopathy is estimated to be 0.4%-6.4% [2]. To the best of our knowledge, the recurrence rate of vascular abnormalities associated with NF1 has not been definitively determined due to the lack of data. The present case was unique in that the massive hematoma presented shortly after the patient received acupuncture treatment and there were no other identifiable causes, suggesting a risk of malformed vessel rupture in NF1 patients after minor injury/trauma such as those associated with acupuncture therapy.

In the past, open surgery was considered the gold-standard treatment for pseudoaneurysms. Nowadays, less invasive interventional therapy has become more popular [10]. Therapeutic embolization involves the intentional endovascular occlusion of an artery or vein [11]. PVA leads to permanent occlusion by adhering to the vessel wall, causing stagnation of flow [12], and has been widely used since it was first introduced as an intravascular embolization agent in 1974. In our case, interventional arterial embolism by injecting PVA stopped active bleeding, stabilized the patient, and played a decisive role in bridging her to eventual surgical resection.

## Conclusions

We presented a case of a female NF1 patient with a massive hemorrhage from a malformed tumor vessel after undergoing acupuncture therapy. This case report suggests that minor injuries such as those associated with acupuncture could cause severe bleeding in patients with vascular malformation related to NF1. Interventional angiography and endovascular embolization were effective in localizing the culprit vessel and stopping active bleeding in our patient.
